# Supplementary material for: Mixing of a binary passive particle system using smart active particles
Source: Sci Rep. 2025 Dec 20;16:3174. doi: 10.1038/s41598-025-33076-6 (PMC12830705; doi:10.1038/s41598-025-33076-6)
Supplement: Supplementary file 1 — Supplementary Information. [file 41598_2025_33076_MOESM1_ESM.pdf]

# Supplementary Information
## Mixing of a binary passive particle system using smart active particles

**Thomas Jacob[1,2], Siddhant Mohapatra[1], Rajalingam A[1], Sam Mathew[3], and Pallab Sinha Mahapatra[1,*]**

[1]Department of Mechanical Engineering, Indian Institute of Technology Madras, 600036 Chennai, India.
[2]Department of Mechanical Engineering, Mar Athanasius College of Engineering, 686666 Kothamangalam, India.
[3]Fachbereich Physik, Freie Universität Berlin, Arnimallee 14, 14195 Berlin, Germany.
[*]email: pallab@iitm.ac.in

## SI-1: Training architecture

A reinforcement learning (RL) framework has been employed to train the agent controlling the smart active particles (SAPs). The three SAPs move in the domain with a fixed self-propulsion velocity of $r$ distance units per unit time ($r$ being the radius of the SAP). It is the orientations of these particles that undergo a change after predefined time steps, and are controlled by a neural network (NN) for which an MLP policy network with multiple hidden layers is used. As the training is carried out using Reinforcement Learning (RL), there are two networks: a policy network and a value network. The policy determines the action to be taken at a certain state, and the value network quantifies the effect of that action.

The RL-based training is implemented for the policy using *stable-baselines3* library. This library offers Proximal Policy Optimisation (PPO), an RL algorithm used in the current work to update the parameters of both the policy and the value networks. During the training process, the PPO optimises a combined loss function (Eq. 1) which involves the policy loss (Eq. 2), value loss (Eq. 3) and entropy loss (Eq. 4).

$$\mathscr{L}_{\text{total}} = \mathscr{L}_{\text{policy}} + c_1 \cdot \mathscr{L}_{\text{value}} - c_2 \cdot \mathscr{L}_{\text{entropy}} \tag{1}$$

where, $\mathscr{L}_{\text{policy}}$ is the clipped surrogate policy loss, $\mathscr{L}_{\text{value}}$ is the value function (critic) loss, $\mathscr{L}_{\text{entropy}}$ is the entropy bonus for exploration, and $c_1$ & $c_2$ are value function coefficient and entropy coefficient, respectively.

$$\mathscr{L}_{\text{policy}} = -\mathbb{E}_t\left[\min\left(r_t(\theta)\hat{A}_t,\ \text{clip}\left(r_t(\theta), 1-\varepsilon,\ 1+\varepsilon\right)\hat{A}_t\right)\right] \tag{2}$$

where, $r_t(\theta) = \frac{\pi_\theta(a_t|s_t)}{\pi_{\theta_{\text{old}}}(a_t|s_t)}$ is the probability ratio, $\pi_\theta(a_t|s_t)$ is the probability of taking action $a_t$ in state $s_t$ under the current policy with parameters $\theta$, $\pi_{\theta_{\text{old}}}(a_t|s_t)$ is the probability under the old policy before the current update, $\hat{A}_t$ is the estimated advantage at time $t$, and $\varepsilon$ is the PPO clipping parameter.

$$\mathscr{L}_{\text{value}} = \mathbb{E}_t\left[\left(V_\theta(s_t) - \hat{R}_t\right)^2\right] \tag{3}$$

where, $V_\theta(s_t)$ is the value function (state-value estimate) under parameter $\theta$, and $\hat{R}_t$ is the estimated return at time $t$.

$$\mathscr{L}_{\text{entropy}} = \mathbb{E}_t\left[\mathscr{H}\left[\pi_\theta(\cdot|s_t)\right]\right] \tag{4}$$

where, $\mathscr{H}\left[\pi_\theta(\cdot|s_t)\right]$ is the entropy of the policy's action distribution at state $s_t$.

The entropy coefficient enables the agent to explore the domain more extensively while avoiding determinism. The discount factor helps the algorithm choose between immediate rewards and long-term rewards. A larger discount factor promotes future rewards but can slow down the learning process. The learning rate is crucial in controlling the updates to the policy parameters, with larger values leading to instabilities.

In the PPO algorithm, a bunch of "experiences" is collected during the agent's interaction with the environment using the current policy. This experience consists of state, action, reward, and value. Using these experiences, the advantage estimates are calculated. Although there are two variants of PPO, one with an adaptive KL penalty and the other with a clipped surrogate objective, *stable-baselines3* uses the latter due to its simplicity and lower computational resource requirements.

**Table S1.** Default parameter set for PPO in the *stable-baselines3* library

| Parameter | Values |
|---|---|
| learning rate(lr) | $3 \times 10^{-4}$ |
| Entropy coefficient | 0 |
| Discount factor ($\gamma$) | 0.99 |
| Clip range | 0.20 |
| n_steps | 2048 |
| batch_size | 64 |
| n_epochs | 10 |
| ent_coef | 0 |
| vf_coef | 0.50 |
| gae_lambda | 0.95 |
| clip_range_vf | None |
| normalize_advantage | True |
| max_grad_norm | 0.5 |
| use_sde | False |
| sde_sample_freq | -1 |
| target_kl | None |
| stats_window_size | 100 |
| tensorboard_log | None |
| policy_kwargs | None |
| verbose | 0 |
| seed | None |
| device | 'auto' |
| _init_setup_model | True |

For implementing the PPO-based RL training approach, a set of hyperparameters must be preset before training. The *stable-baselines3* library uses default values if not explicitly specified. The simulations have been carried out using the default hyperparameters (refer to Table S1). The input values to the policy are represented as a one-dimensional array. Therefore, the x and y coordinates data of both active and passive particles are concatenated into a single dimension of the form $x_1, y_1, x_2, y_2, \cdots x_n, y_n$, and passed on as input to the policy. The position data of the boundary particles are not included as they remain unchanged across the training. The algorithm in its default configuration collects 2048 experiences before updating the policy with a batch size of 64. For carrying out the initial simulations with default parameters, the SAPs' run duration $\delta = 2 \times 10^3$, and tumble angle step $\Gamma = \pi/2$ are set. Using the default hyperparameter values for PPO as defined in Table S1, the policy fails to learn and optimise.

To choose a NN architecture, the value of $\Gamma$ is initially set to $\pi/2$, and that of $\delta$ to $2 \times 10^3$ and training is carried out with different neural networks for the same initial conditions of the particle system. During training, the agent interacts with the environment over multiple episodes. A maximum episode length is also set, and the episode restarts in case the agent is able to optimise the system to a peak mixing index before reaching the maximum length. This helps the system undergo training over the maximum number of episodes, keeping the total number of agent-environment interactions fixed ($\approx 10^6$). To determine if the agent is undergoing a favourable parameter update during training, the length of episodes required for the agent to achieve a mixing index of 0.99 is plotted. In case of favourable parameter updates, the episode length should decrease as the training progresses.

## SI-2: Algorithm of the training code

The algorithm of the simulation code is elucidated in Fig. S1. The passive particles are initially distributed in the domain such that one species occupies the upper half, while the other species occupies the lower half. The active particles are distributed uniformly at random across the domain. The code for training and testing has two sub-routines: the Environment API written in *Python3* using the OpenAI Gym interface, along with the associated user-defined functions, and the particle dynamics sub-routine written in *C*. The *ctypes* library of *Python* is used for communication between the two sub-routines. For training the RL agent, the Gym Environment interface, a standard template defined by OpenAI Gym, is used. The environment in the code is defined under the class `ParticleMixer`. This class manages the environment and its dynamics using several API

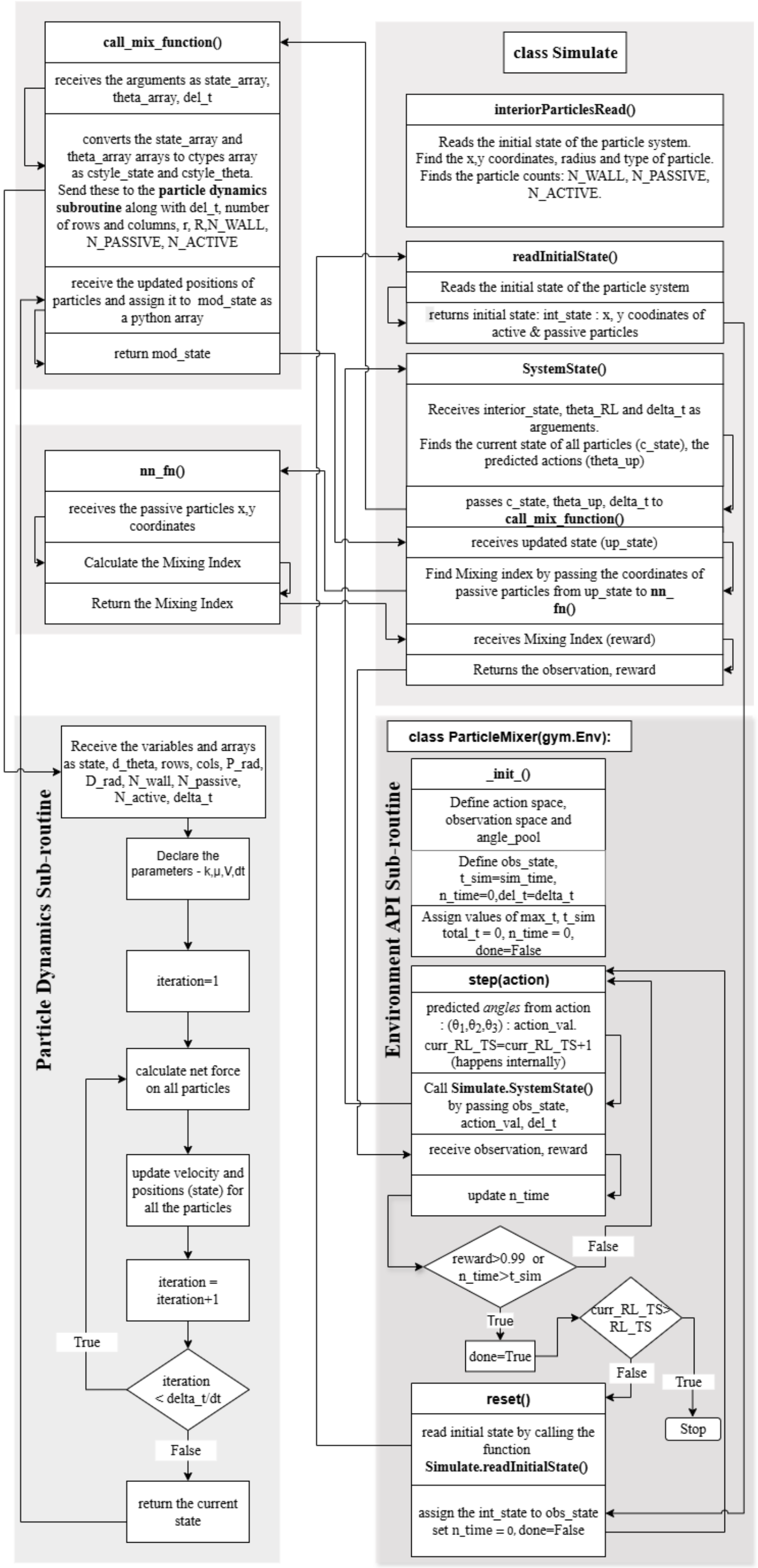


**Figure S1.** The algorithm of the training code has two sub-routines: the Environment API handles the training of the agent, whereas the particle dynamics sub-routine takes care of the actual particle movements in the environment. The former is written in *Python3* and uses the OpenAI Gym interface, while the latter is written in *C* and is coupled to the Environment API through *ctypes*.

methods, which are outlined below.

- `_init_` - a constructor which defines the `action_space`, `observation_space` and values of other gym parameters and variables required for the training.
- `step()` - updates the environment from the old state to the new state with return values as observation, reward, termination status of environment, and additional information.
- `reset()` - resets the environment to its initial state. Called before `step()` during the beginning of the training episode.
- Additional functions such as `render()` and `close()` perform any rendering or clean-up tasks. If not utilised, the placeholder `pass` is specified.

The custom environment used for the training is defined under the `ParticleMixer` class, which inherits from `gym.Env`, and an instance of the custom environment class is created using the command `env = ParticleMixer()`. The Gym environment observes the state of the system, determines the actions corresponding to that state, and receives rewards for those actions. When creating the environment using Gym, a defined format must be followed. As soon as the environment is created, the `_init_` initialises the action space, observation space and several other variables. The `action_space` defines the possible actions that can be predicted by the agent (here, the directional motion for the active particles, which is Multi-discrete in nature). Actions for each of the SAPs are in the range $[0, 2\pi)$ in steps of $\Gamma$ (the lowest factor of tumbling angle). $\Gamma = \pi/2$ leads to four possible actions, i.e. North, South, East and West. The `observation_space` defines the minimum and maximum values possible for the observations. It can be defined in terms of `DOMAIN_RAD`, which represents the distance from the centre of the domain to the centre of the boundary particles. In the current work, the domain has been placed entirely in the first quadrant due to the usage of the *ReLU* activation function for the Neural Network.

In the current implementation, the simulation begins by calling the function `Simulate.interiorParticlesRead()`, which initialises several variables and arrays necessary for the training module. When training the model, the information flow begins with `reset()`, where the initial observation is defined as a 1D array representing the positions of both active and passive particles. Following this, the agent (NN) decides on an action based on the observation. The action is now passed to the `step()` function. Here, the variables $(\theta_1, \theta_2, \theta_3)$ are updated based on the action. The action, observation, and `delta_t` values are then passed to an interim function `SystemState()` under the class `Simulate`, which then transmits the requisite variables and arrays to the function `call_mix_function()`. This function converts the arrays and variables into C-style arrays and transmits them to the integrated C subroutine (particle dynamics subroutine) for simulating particle dynamics based on overdamped Langevin equations. The particle dynamics sub-routine, after simulating the interactions between the active, the passive and the wall particles for a duration of `delta_t`, reverts the updated position data of all the interior particles back to `call_mix_function()`, where it is converted to a Pythonic array (`mod_state`). This Python-style array is then passed to the `SystemState()` function, which subsequently calls the `nn_fn()` function to compute and return the mixing index using the position data of the passive particles. The mixing index also serves as the reward for the actions undertaken by the agent. The function `SystemState()` then transmits the latest position data (observation) and the reward to the `step()` function in the Environment API sub-routine. This completes one interaction between the agent and the environment. Such interaction occurs cyclically, resulting in a collection of experience tuples (formed from state-action-reward), based on the rollout length (in PPO, `n_steps`). In the background of the PPO engine, all experience tuples are stored in a buffer. The total number of agent-environment interactions is explicitly set and represented by `RL_TS` in the algorithm.

The model used for training is defined by including the policy `MlpPolicy`, the custom Gym Environment `env`, custom neural network hidden layer structure `policy_kwargs`, and `hyperparameters`. In the current implementation, a shared structure is used for both policy and value networks. The default values of the hyperparameters in the *stable-baselines3* library are tabulated in Table S1.

The `done` variable defined in the Environment API subroutine determines the end of the episode, when either of the following conditions holds true: the system achieves a mixing index $\chi > 0.99$ or the time taken for the training exceeds the maximum threshold set (`n_time>t_sim`). To quantify the training efficiency, the length of each training episode is stored along with the final reward.

## SI-3: Selection of hyperparameters and policy architecture

The most critical among the hyperparameters is the learning rate $lr$. It decides the degree of modulation occurring in the model being trained. Values that are too high can cause learning to diverge from the path to optimality, whereas values that are too low, albeit stable, can increase the time required to reach the optimal solution. Setting the run duration of the SAPs (also the period of interaction between agent and environment) $\delta = 2 \times 10^3$, and minimum tumble angle $\Gamma = \pi/2$ following the results from the run-and-tumble particle simulations, different learning rates and hidden layer architecture pairs tabulated in Table S2 are

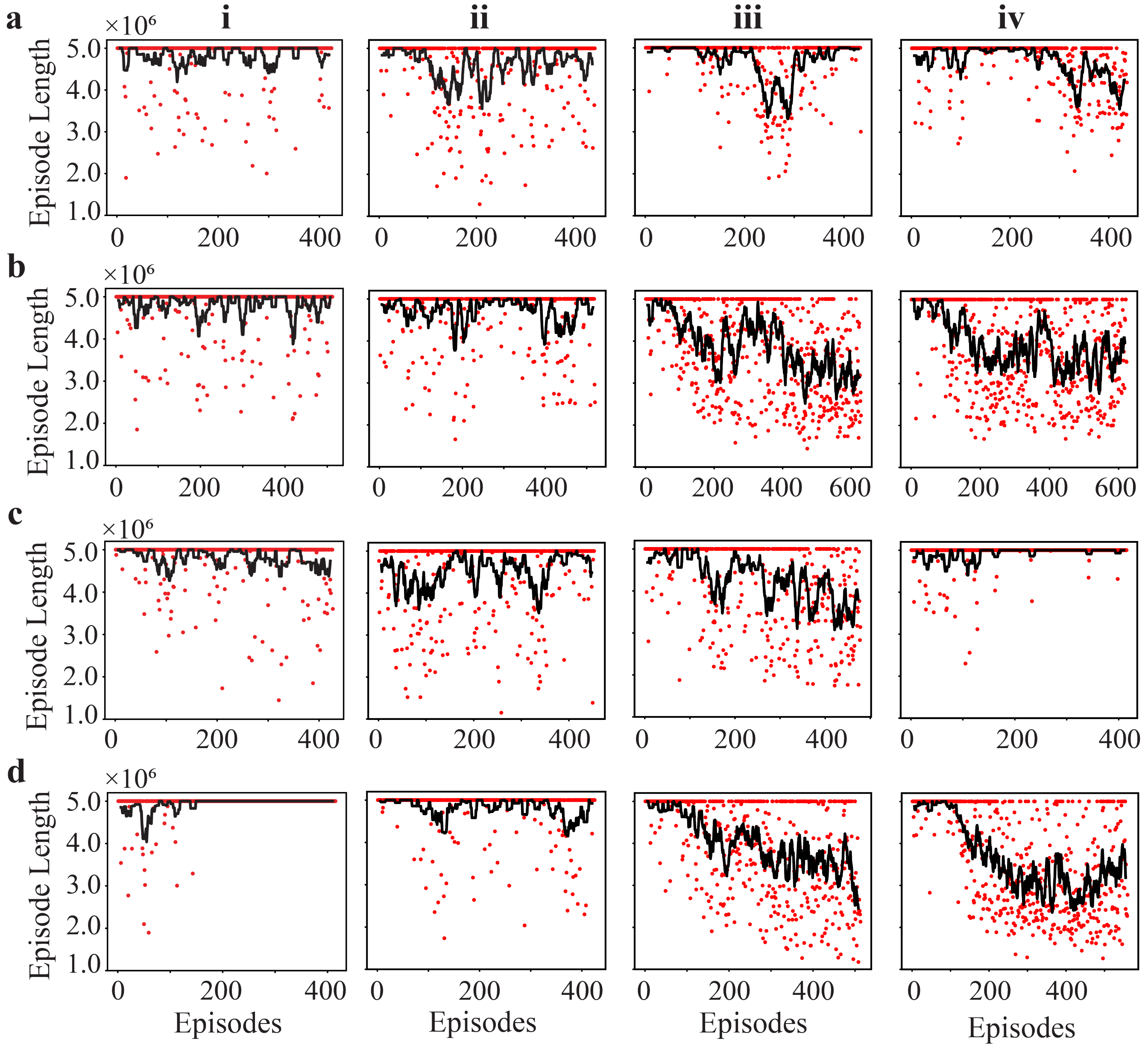


**Figure S2.** The variation of episode length is plotted as red dots across episodes as the training proceeds for all the combinations of learning rate $lr$ and hidden layer architectures tested. The black solid line represents a moving window average of the episode length. Rows (a) through (d) represent the hidden layer configurations for the policy - $(256,128,64)$, $(512,256,64)$, $(256,128,128,64)$, and $(512,256,128,64)$, respectively; while columns (i) through (iv) represent different learning rates - $3\times10^{-4}$, $10^{-5}$, $10^{-6}$, and $5\times10^{-7}$, respectively. The remaining hyperparameters have been set to default values as mentioned in Table S1. A learning rate of $10^{-6}$ is found to be suitable for carrying out the simulations. ($\delta = 2\times10^{3}$, and $\Gamma = \pi/2$).

**Table S2.** Parameter space for the simulations

| Parameter | Values |
|---|---|
| learning rate(lr) | $3\times10^{-4}$, $10^{-5}$, $\mathbf{10^{-6}}$, $5\times10^{-7}$ |
| hidden layer (NN) sizes | $(256,128,64)$,$(\mathbf{512},\mathbf{256},\mathbf{64})$, $(256,128,128,64)$, $(512,256,128,64)$ |
| Period of Interaction ($\delta$) in time steps | $0.5\times10^3$,$1\times10^3$, $\mathbf{2\times10^3}$,$3\times10^3$ |

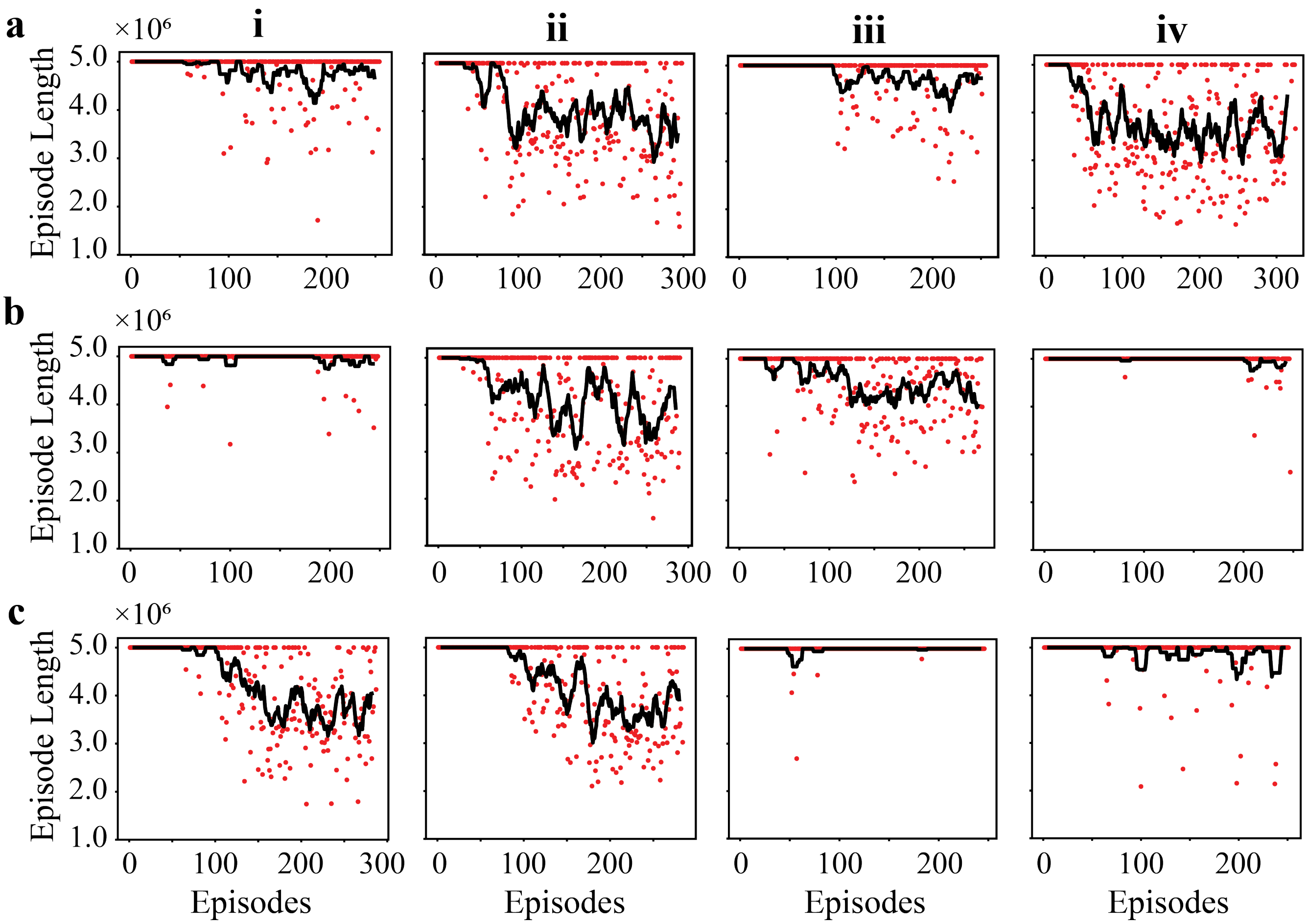


**Figure S3.** The variation in episode length across training episodes is plotted as red dots for different $\Gamma$ and NN hidden layer configurations. For all the cases, $\delta$ is fixed at $10^3$ and learning rate at $lr = 10^{-6}$. The solid black line represents a moving average of the episode length. Rows (a) through (c) correspond to $\Gamma$ values of $\pi/2$, $\pi/4$, and $\pi/8$, respectively; while columns (i) through (iv) refer to hidden layer configurations for the policy - $(256,128,64)$, $(512,256,64)$, $(256,128,128,64)$, and $(512,256,128,64)$, respectively

explored as part of the preliminary simulations. All other hyperparameters required for PPO are set to default values as listed in Table S1. To visualise the efficacy of learning, the length of each training episode is plotted against the episode number. For an agent which is learning (i.e., moving closer to the optimality criteria with each episode), the episode length should decrease as the training progresses. Considering a wide range of learning rates $lr$ (from a default value of $3\times10^{-4}$ to $5\times10^{-7}$) and a pool of hidden layers NN architecture (see Table S2), Fig. S2 illustrates the learning efficacy for all combinations, keeping $\delta$ and $\Gamma$ fixed at $2\times10^3$ and $\pi/2$, respectively. It is evident from the reduction in episode lengths during training that a lower learning rate is favourable to the training process. Therefore, a learning rate of $lr = 10^{-6}$ is found to be sufficient for training the RL agent for the current study.

After choosing the learning rate, the next step is to select an appropriate neural network architecture for the RL agent. To choose an architecture which works well with a range of $\delta$ and $\Gamma$ values, simulations are carried out for all possible combinations mentioned in Table S2. In all cases, the training is carried out for $1.2 \times 10^6$ interactions between the agent and the environment.

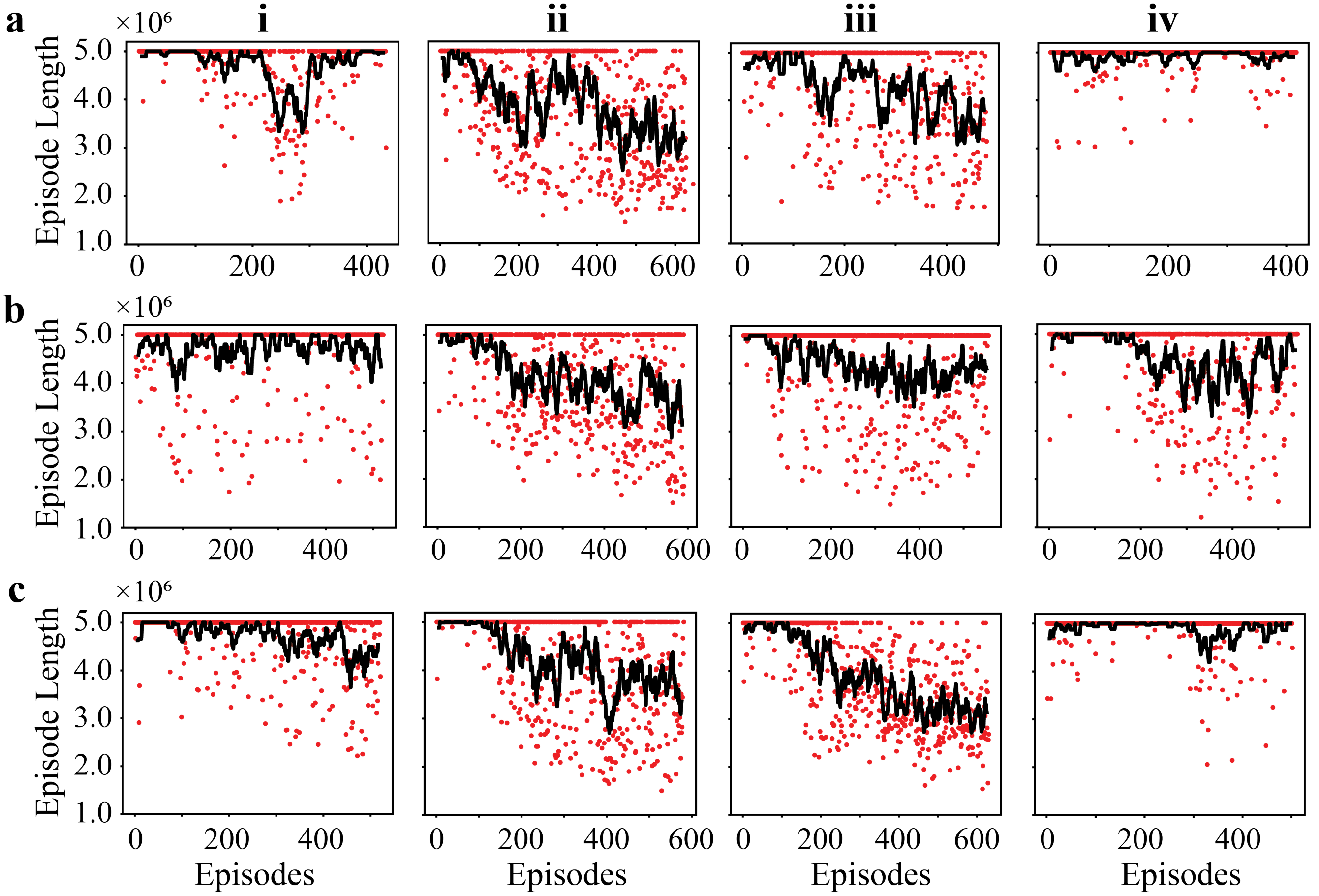


**Figure S4.** The variation in episode length across training episodes is plotted as red dots for different $\Gamma$ and NN hidden layer configurations. For all the cases, $\delta$ is fixed at $2 \times 10^3$ and learning rate at $lr = 10^{-6}$. The solid black line represents a moving average of the episode length. Rows (a) through (c) correspond to $\Gamma$ values of $\pi/2$, $\pi/4$, and $\pi/8$, respectively; while columns (i) through (iv) refer to hidden layer configurations for the policy - $(256, 128, 64)$, $(512, 256, 64)$, $(256, 128, 128, 64)$, and $(512, 256, 128, 64)$, respectively.

The variation of the episode lengths across episodes for different $\delta$, $\Gamma$, and NN hidden layer configuration are illustrated in Figs.S3 through S5 for $\delta = 10^3$, $\delta = 2 \times 10^3$ and $\delta = 3 \times 10^3$ respectively. Each of these figures elucidates the learning efficacy with various combinations of NN hidden layer configuration and $\Gamma$. If $\delta$ is too low ($\delta = 0.5 \times 10^3$), the learning underperforms irrespective of the complexity of the NN architecture and $\Gamma$, and hence, has not been shown here for brevity. However, an increase in $\delta$ to $\delta = 10^3$ and $\delta = 2 \times 10^3$, better performance is observed with a hidden NN architecture of layer sizes 512, 256, and 64 (see Figs. S3 and S4). It shows a decreasing episode length (as indicated by the solid black line, which represents a moving average of the episode length data shown by the red dots) as training progresses, striking a balance between an increasingly complex neural network structure and a larger action space (due to a finer $\Gamma$). A larger interaction period ($\delta = 3 \times 10^3$) is found to worsen learning efficacy (see Fig. S5), and therefore, is not preferable. On the other hand, for the selected hidden layer configuration (512, 256, and 64), $\Gamma$ is observed to have a marginal effect on the learning performance of the agent. Therefore, $\Gamma = \pi/2$ is chosen to reduce the action space, while not compromising on the learning characteristics.

## SI-4: Residence time of passive particles in the presence of RT particles

When the binary passive system is mixed using run-and-tumble (RT) particles, the passive particles are observed to reside longer near the domain boundary, due to the pushing action by the RT particles situated close to the domain centre. Once the passive

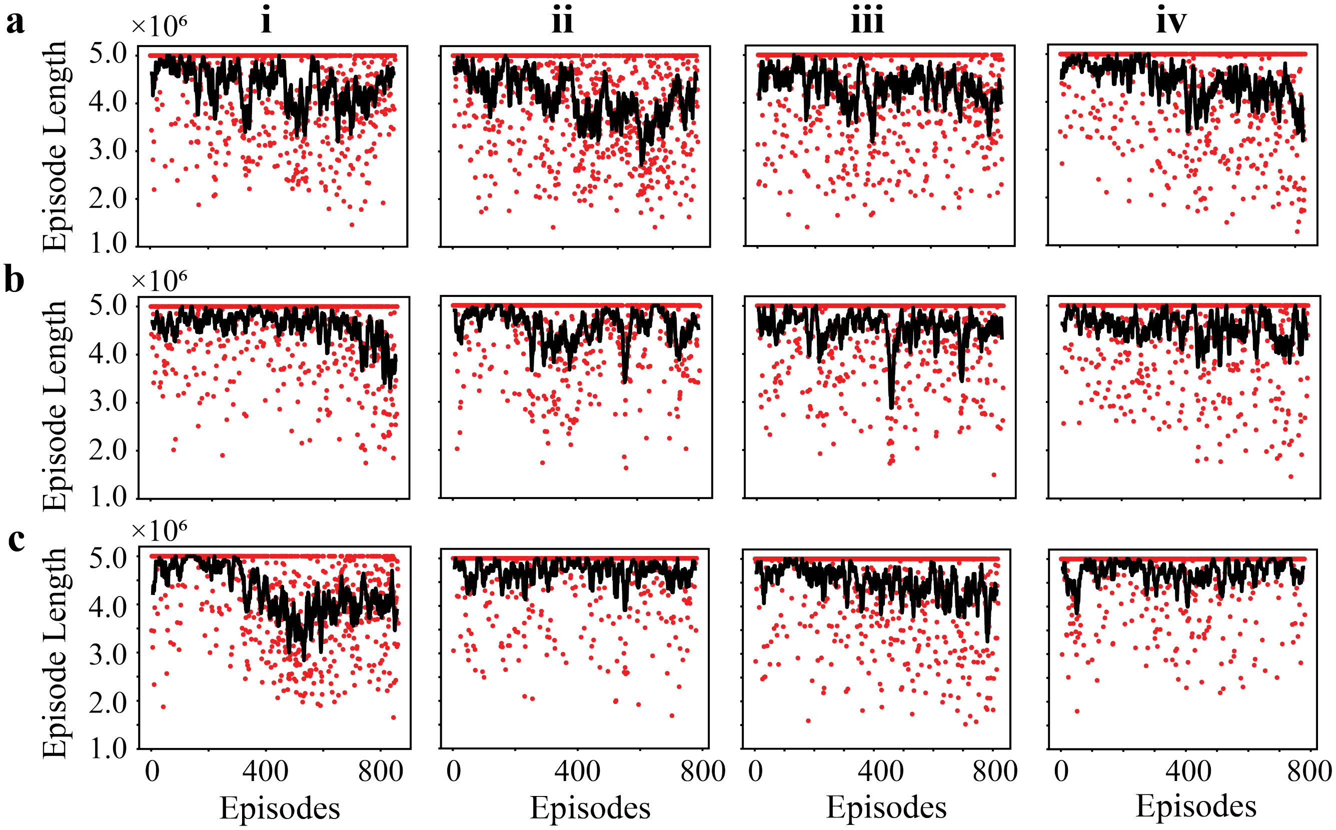


**Figure S5.** The variation in episode length across training episodes is plotted as red dots for different $\Gamma$ and NN hidden layer configurations. For all the cases, $\delta$ is fixed at $3\times10^3$ and learning rate at $lr = 10^{-6}$. The solid black line represents a moving average of the episode length. Rows (a) through (c) correspond to $\Gamma$ values of $\pi/2$, $\pi/4$, and $\pi/8$, respectively; while columns (i) through (iv) refer to hidden layer configurations for the policy - $(256,128,64)$, $(512,256,64)$, $(256,128,128,64)$, and $(512,256,128,64)$, respectively.

particles are moved close to the boundary, they can mostly be pushed along the boundary, unless an active particle wedges itself between the passive particle and the wall particles. To quantify the residence time of the passive particles, the circular domain is divided into concentric regions of radial width $2r$ (where $r$ is the radius of the passive particle). The concentric regions (excluding the central area) are numbered from 1 to 6, starting from the outermost band. A sketch of the division of the domain is shown in Fig. 8(b) of the manuscript. Figure S6 demonstrates the probability distribution of residence time $t_r$ in the different bands, considering a single representative simulation data. It is evident that bands 1 and 2, near the wall, display residence time orders of magnitude higher than those close to the domain centre.

## SI-5: Analogous modelling of mixing dynamics

A transition from a clockwise to a counter-clockwise motion of the passive particles is observed along the radius towards the centre during mixing induced by smart active particles. The active particle dynamics appear to behave similarly to those of an elliptical-shaped wall placed at an offset from the domain centre and inducing a flow in the passive environment through a constant surface speed. To check the veracity of this hypothesis, a computational model is developed and analysed using the ANSYS Fluent ™software package to replicate similar velocity patterns on a stratified liquid system during mixing. The geometry consists of a circular outer domain with an inner elliptical wall as shown in Fig. S7. The ellipse is located at an offset towards the left of the domain centre, roughly demarcating the operational area of the active particles. Two liquids (liquid 1 and liquid 2) with the same properties (density of $998.2 kgm^{-3}$ and viscosity of $1.003\times10^{-3} Pa\cdot s$) are placed such that the interface coincides with the minor axis of the ellipse. Both the ellipse and the circular wall possess defined absolute velocities, and the no-slip boundary condition has been preset. Therefore, the ellipse effectively acts as a vortex, inducing motion to the fluid due to viscous effects. The speed of the ellipse is kept constant along the surface, and the analysis is performed for different values of the ratio $v_r = v_c/v_e$ of the outer circular confinement $v_c$ to that of the speed of the inner elliptical wall $v_e$.

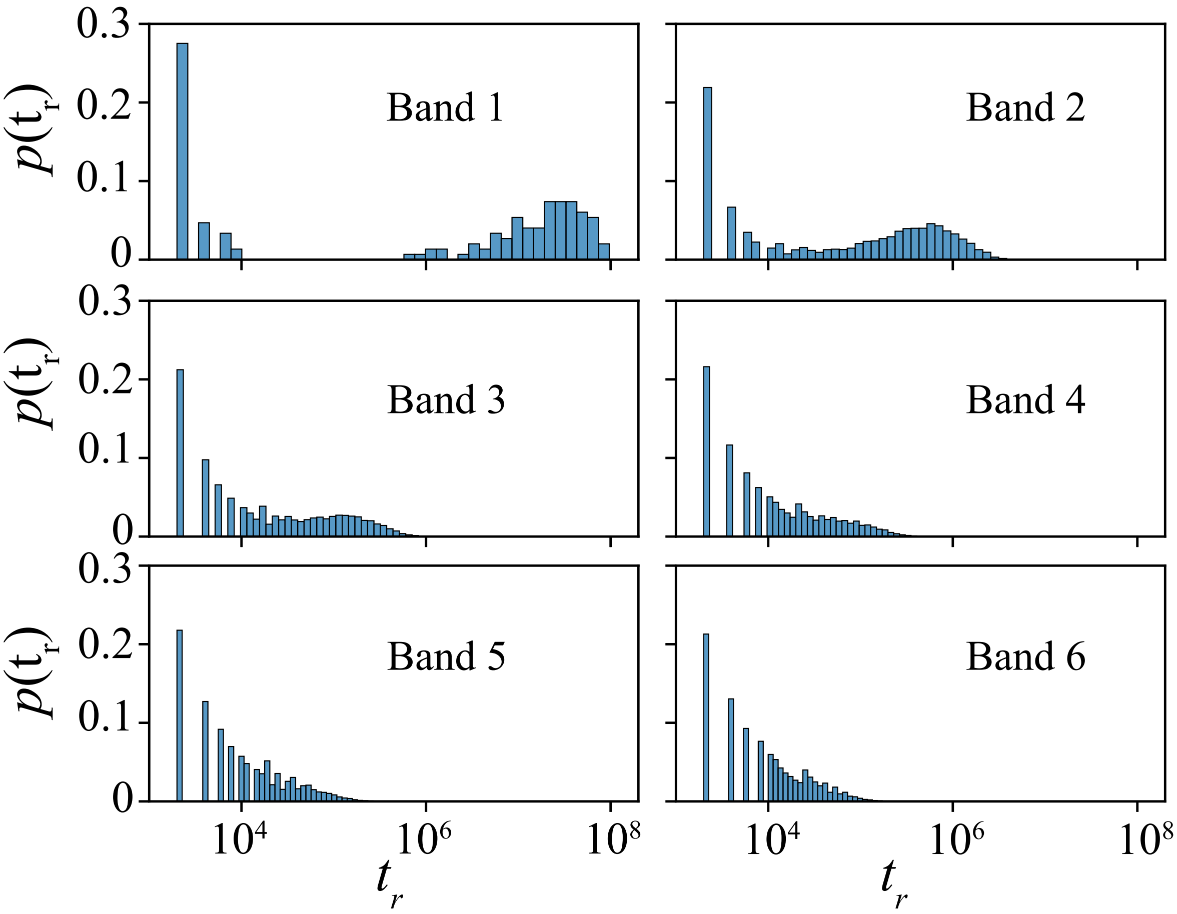


**Figure S6.** The circular domain is divided into six concentric regions (excluding the central area), numbered $1-6$, starting from the outermost band, with each concentric band having a radial width of $2r$ ($r$ being the radius of the passive particle). The probability distribution of residence time ($t_r$) of the passive particles in the presence of run-and-tumble particles is plotted for each of these bands. The passive particles are observed to have higher residence time in bands 1 and 2, which are closer to the domain wall, compared to the domain centre. (Note: The data presented here corresponds to a representative simulation of mixing effected by RT particles.)

The simulations are solved for the steady-state governing equations, assuming incompressible Newtonian flow, an isothermal setup and neglecting viscous dissipation. A coupled algorithm is employed for the pressure–velocity coupling, utilising the PRESTO scheme for pressure interpolation. The convective terms in the momentum equations are discretised using the second-order upwind scheme. For the phase tracking, the Volume of Fluids (VOF) method with compressive interface capture is employed to ensure sharp reconstruction of the fluid-fluid interface. The domain is divided into a uniformly structured grid comprising 91000 elements, balancing grid resolution and computational cost. The convergence criteria for the simulations are set such that the residuals for all the governing equations (see Eqs. 5, 6, 7) fall below $10^{-4}$.

Continuity equation:

$$\nabla \cdot \mathbf{v} = 0 \tag{5}$$

Momentum equation:

$$\rho_l (\mathbf{v} \cdot \nabla)\mathbf{v} = -\nabla p + \mu \nabla^2 \mathbf{v} \tag{6}$$

Phase transport equation:

$$\mathbf{v} \cdot \nabla \alpha = 0 \tag{7}$$

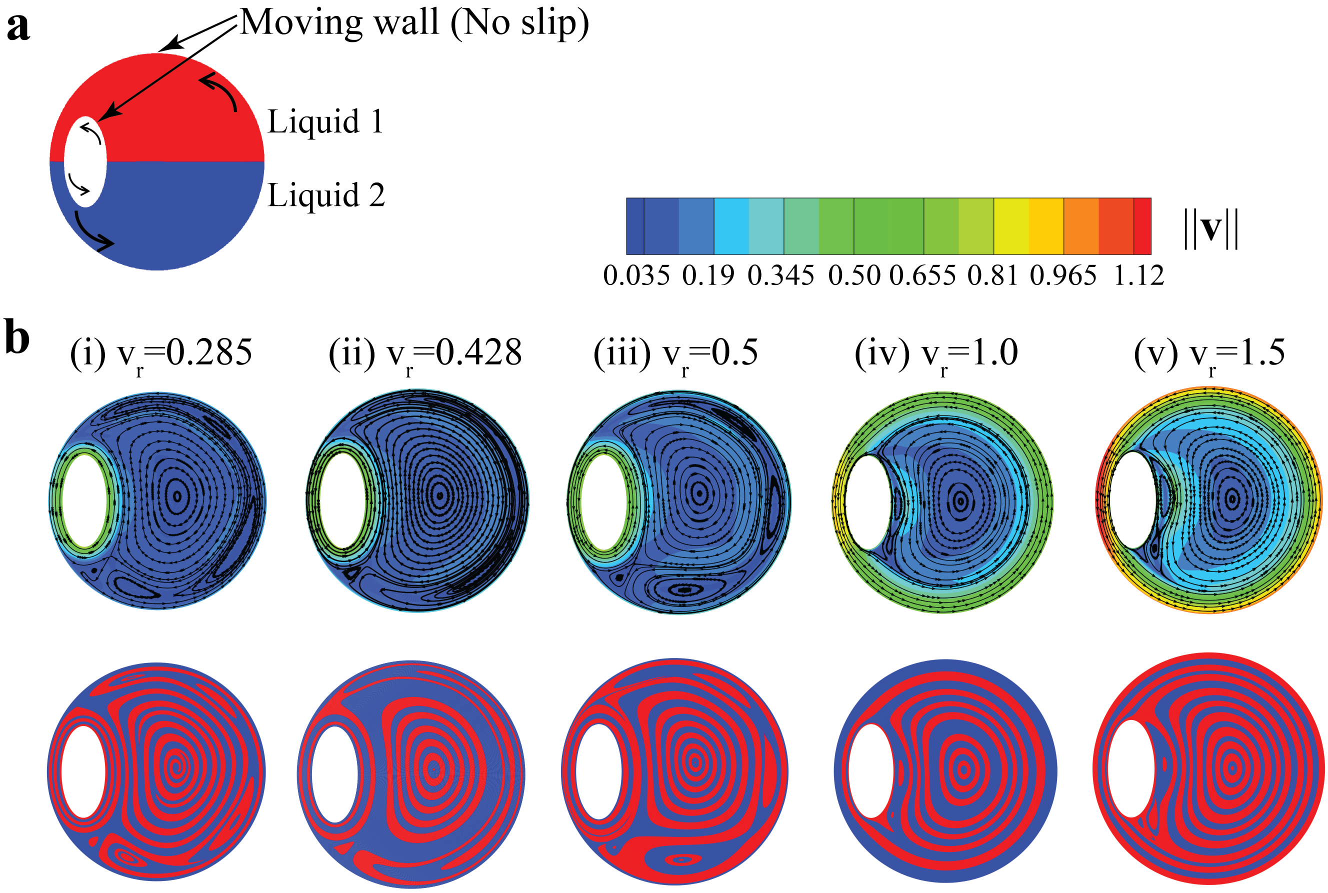


**Figure S7.** An analogous model is defined to demonstrate the mixing of two stratified liquid layers using a moving circular outer wall and an eccentrically located elliptical inner wall with a surface velocity. Panel (a) illustrates the initial configuration of the system, along with the direction of motion of the inner and the outer walls (both walls move with a counter-clockwise handedness). Panel (b) elucidates the velocity distribution and streamlines (top sub-panel) as well as the phase distribution (bottom sub-panel) of the two fluids after attaining steady state during mixing. Cases (i) through (v) represent the steady state quantities for the system with a variation in the speed ratio $v_r$ (defined as the ratio of the speed of the outer circular wall to the speed of the inner ellipse). (Note: The colour bar denotes the magnitude of velocity of a fluid element, $||\mathbf{v}||$. The speed of the inner elliptical wall is fixed at $v_e = 0.7 m/s$.)

where $\mathbf{v}$, $\rho_l$, $p$, $\mu$ and $\alpha$ represent the velocity vector, the density of the liquid, the pressure, the dynamic viscosity and the volume fraction, respectively. All the quantities are in SI units.

Figure S7 illustrates the initial configuration of the computational domain (panel (a)) together with the distribution of phases, velocity variation, and streamlines for the three cases considered (panel (b)). The results indicate that the speed ratio between the ellipse and the circle has a strong influence on the velocity distribution, and that the phase distribution changes accordingly. The velocity contours (see top sub-panel of Fig. S7(b)) reveal that the movement of the walls drives the fluid motion through viscous shear. The placement of the ellipse is such that there is a narrow space between the inner and the outer walls to the left of the ellipse. The narrow gap accelerates the fluid passing through it, contributing to the overall mixing. As the inner and the outer walls rotate in the same direction, the fluid layer adjacent to the wall also moves in the same direction due to the viscous effect. However, due to the shear effect, vortices are also observed between the two layers. For a lower velocity ratio (see Figs. S7(b-i), S7(b-ii) and S7(b-iii)), the inner fluid layers display motion in a direction opposite to that of the peripheral layers, and the vortices appear in between these layers. For higher $v_r$ (see Figs. S7(b-iv) and S7(b-v)), the streamlines of the fluid layers align with the directions of both ellipse and circle and the position of the vortex is shifted closer to the ellipse. The overall motion of the two liquids bears a close resemblance to the movement of the passive particles in the binary passive system.

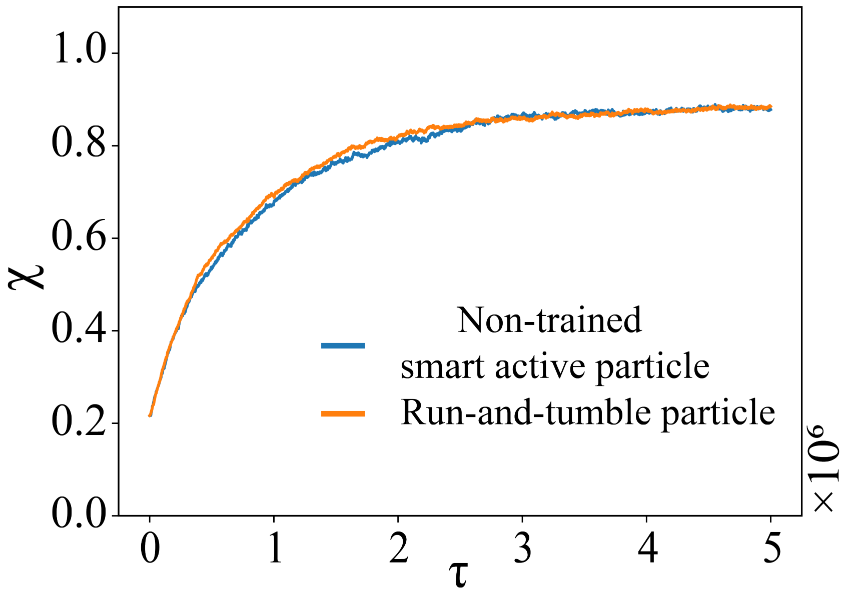


**Figure S8.** Comparison of the temporal variation in mixing performance of active particles controlled by a non-trained RL agent (NTSAPs; blue solid line) and active particles executing run-and-tumble dynamics (RTPs; orange solid line). A mixing index $\chi$ is used to quantify the state of mixing in the passive system. In case of the NTSAPs, $\delta = 2 \times 10^3$, $\Gamma = \pi/2$, and a NN architecture with hidden layer sizes of 512, 256, and 64. In case of the RTPs, mean run duration is $\tau_m = 2 \times 10^3$ and follows an exponential distribution, while tumbling angles are uniformly distributed across $[0, 2\pi)$. All other parameters for the particle dynamics simulations are kept unchanged across the two cases.

## SI-6: Run-and-tumble particles vs non-trained SAPs

The smart active particles (SAPs) in the present study, when controlled by a non-trained RL agent, can mimic the motion of run-and-tumble particles (RTPs) with a fixed run duration and an instantaneous tumble. To test this hypothesis, simulations were conducted separately with RTPs and non-trained SAPs. The RTPs are considered to have run-and-tumble motion (ballistic runs with instantaneous tumbles) with a run duration sampled from an exponential distribution (first moment being $\tau_m = 2 \times 10^3$) and tumbling angle sampled from a uniform random distribution across the range $[0, 2\pi)$. For the non-trained SAPs, $\delta = 2 \times 10^3$ and $\Gamma = \pi/2$. All the other parameters used in the particle dynamics simulations are kept invariant across the two cases. The mixing index $\chi$ is calculated for both cases, averaged across fifty realisations. As expected, similar mixing performance is observed for both cases (refer to Fig. S8). This implies that the non-trained agent transmits random action values, which belong to the same paradigm as that of active particles executing RT motion.

## SI-7: Dependence of initial particle distributions

The training of the neural network in the current work is carried out using a single set of initial positional configurations of the active particles. To highlight the compatibility of the agent with any initial positional configuration, four systems with a difference in the initial conditions have been selected (see Fig. S9(a)). In all the instances, the agent with the characteristics as highlighted (bold font) in Table S2, is noted to converge towards a favourable parameter set, as seen from the reducing episodic length with the progression in training (see Fig. S9(b)). The input comprises the x and y coordinates of particle placements, excluding wall particles, with training spanning 1.2 million interactions between the agent and the environment.

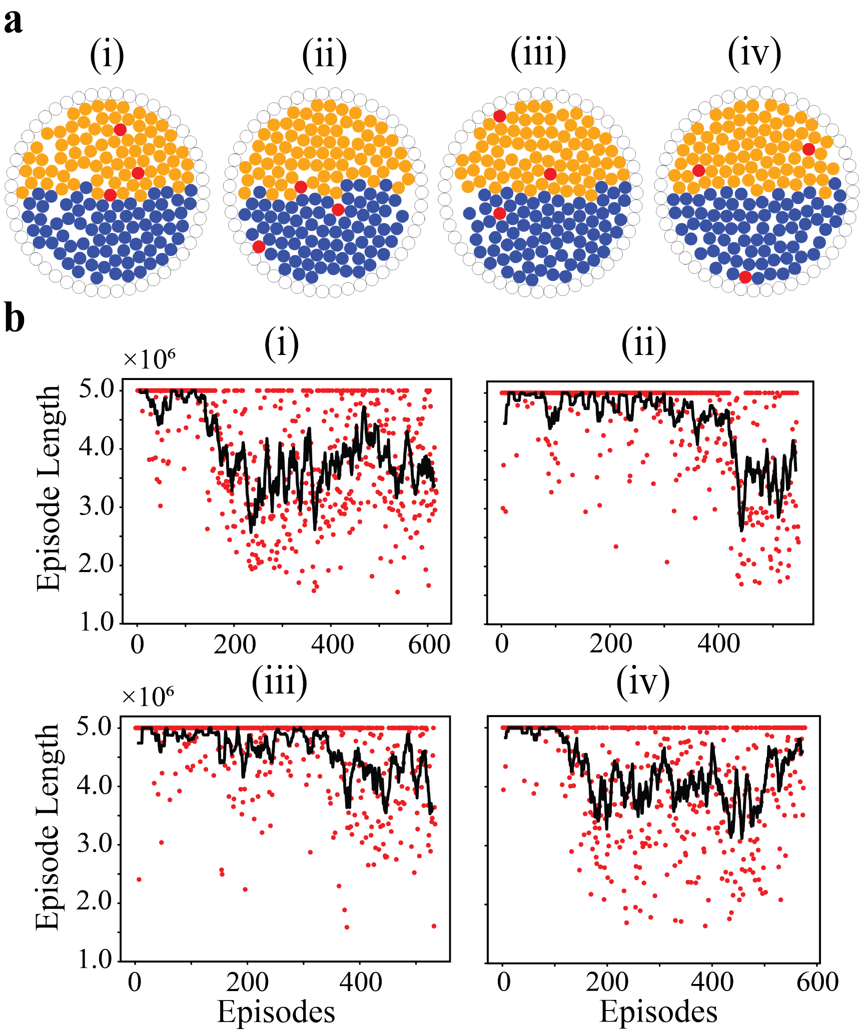


**Figure S9.** The agent controlling the SAPs is trained to mix four different binary passive systems as illustrated in panel (a). The two passive species are coloured blue and orange, while the active particles are coloured red. The parameter set used for the training is tabulated in Table S2. Panel (b) illustrates the training performance pertaining to each of the cases in panel (a), indicating a decrease in episodic length as training progresses in all cases (which points to favourable parametric updates for the agent). The red dots represent the individual episode lengths, while the black solid line presents a moving average of the same.
